# Genome-wide identification and characterization of *JAZ* gene family in upland cotton (*Gossypium hirsutum*)

**DOI:** 10.1038/s41598-017-03155-4

**Published:** 2017-06-05

**Authors:** Wen Li, Xiao-Cong Xia, Li-Hong Han, Ping Ni, Jing-Qiu Yan, Miao Tao, Geng-Qing Huang, Xue-Bao Li

**Affiliations:** Hubei Key Laboratory of Genetic Regulation and Integrative Biology, School of Life Sciences, Central China Normal University, Wuhan, 430079 People’s Republic of China

## Abstract

Plant JAZ (Jasmonate ZIM-domain) proteins play versatile roles in multiple aspects of plant development and defense. However, little is known about the JAZ family in allotetraploid upland cotton (*Gossypium hirsutum*) so far. In this study, 30 non-redundant *JAZ* genes were identified in upland cotton through genome-wide screening. Phylogenetic analysis revealed that the 30 proteins in cotton JAZ family are further divided into five groups (I – V), and members in the same group share highly conserved motif structures. Subcellular localization assay demonstrated that GhJAZ proteins are localized in the cell nucleus. Quantitative RT-PCR analysis indicated that *GhJAZs* display different expression patterns in cotton tissues, and most of them could be induced by Jasmonic (JA). Furthermore, some *GhJAZ* genes are preferentially expressed in cotton ovules and fibers, and showed differential expression in ovules of wild type cotton and fiberless mutant (*fl*) during fiber initiation. GhJAZ proteins could interact with each other to form homodimer or heterodimer, and they also interacted with some JA signaling regulators and the proteins involved in cotton fiber initiation. Collectively, our data suggested that some GhJAZ proteins may play important roles in cotton fiber initiation and development by regulating JA signaling as well as some fiber-related proteins.

## Introduction

Jasmonate (JA, including jasmonic acid and its oxylipin derivatives) is an important phytohormone that regulating many aspects of plant growth, development, and defense^1–6^. Within the signaling cascades that are triggered by JA, the JAZ proteins play a central role. JAZ proteins belong to plant-specific TIFY family that shares a conserved TIFY × G sequence within the ZIM motif^7^. The second defining feature of JAZs is the highly conserved Jas motif, which has a SLX_2_FX_2_KRX_2_RX_5_PY consensus sequence near the C-terminus and is essential for COI1 (JA receptor) interaction^8–13^. Both ZIM domain and Jas domain are required for JAZ-mediated repression of JA responses. The JAZ proteins, substrates of the SCF^COI1^ complex, function as negative regulators to repress diverse JA responses by directly inhibiting various transcriptional factors (TFs)^4, 8–10^. Upon perception of a JA signal, COI1 recruits JAZ proteins for ubiquitination and subsequent degradation through the 26 S proteasome, thereby multiple TFs are relieved from JAZ-mediated repression, allowing them to activate their respective downstream responses^4, 5, 8–19^. Additionally, a model was hypothesized for JA signaling transduction, in which JAZ proteins recruit the general co-repressors TOPLESS (TPL) and TPL-related proteins (TPRs) through an adaptor protein Novel Interactor of JAZ (NINJA) to block the activity of TFs (such as MYC2) in the absence of bioactive JA^17, 20^. Also, some JAZ proteins (such as JAZ8) contain EAR motifs and recruit TPL independently of NINJA^21^. Additionally, MYC2/3/4, the bHLH family TFs, are the best characterized TFs mediated JA signaling, and interact with all JAZs in *Arabidopsis* ^15, 22–26^.

Cotton, which produces the most prevalent natural fibers used in the textile industry, is one of the mainstays of the global economy, and allotetraploid upland cotton (*Gossypium hirsutum*) accounts for more than 90% of cultivated cotton worldwide^27–29^. Cotton fibers are the single-cell trichomes derived from the epidermal layer of the cotton seed coat. Fiber cells undergo several distinctive but overlapping developmental stages: initiation (-2 to 5 days post anthesis (DPA)), elongation (3 to 20 DPA), secondary cell wall deposition (16 to 40 DPA), and maturation (40 to 50 DPA)^30^. It has been proposed that the regulation mechanism of cotton fiber differentiation is similar to that of *Arabidopsis* leaf trichome and root hair differentiation, and TFs also play important roles in regulating fiber initiation and elongation^30–37^.

In the past years, a number of TFs involved in trichome formation have been identified in *Arabidopsis*. GLABRA1 (GL1, an R2R3 MYB transcription factor), GLABRA3 (GL3, a basic helix-loop-helix transcription factor), its homologue ENHANCER OF GL3 (EGL3), and TRANSPARENT TESTA GLABRA1 (TTG1, a WD40-repeat protein) interact each other to control trichome cell fate^33, 38^. These proteins assemble into a trimeric MYB-bHLH-WD protein complex to promote *GL2* expression, thereby regulating the trichome formation^33^. Additionally, JAZ interacts with GL3 and GL1 to regulate JA-mediated trichome initiation in *Arabidopsis* ^39^. Similarly, GhMYB2 and GaMYB23 (GL1 homolog), GhTTG1/GhTTG3 and GaDEL65 (GL3 homolog) are preferentially expressed in initiated fiber cells of cotton and may regulate cotton fiber initiation and differentiation^31–37, 40, 41^. Several R2R3-type MYB TFs also were reported to be involved in cotton fiber initiation. Overexpression *GhMYB25* increases in cotton fiber initiation and leaf trichome number^42^. On the contrary, RNAi-mediated silencing of *GhMYB25-like* transcripts abolishes expansion and elongation of ovule epidermal cells, resulting in the completely fibreless seeds of cotton^34^. Overexpression of *GhJAZ2* inhibited both lint and fuzz fiber initiation and reduced the fiber length, and GhJAZ2 could interact with GhMYB25-like, which may function as a primary transcription repressor during cotton fiber initiation^35^. However, it is unclear whether cotton JAZ proteins interact with each other or with the other key transcription factors for regulating fiber initiation and elongation so far, and needs to be elucidated.

Recently, 28 *TIFY* genes were identified in a diploid cotton species *Gossypium raimondii*, and some of *JAZs* are expressed in cotton fibers^43^. Another study reported 21, 28 and 50 *TIFY* family members in three cotton species (*G*. *arboretum*, *G*. *raimondii* and *G*. *hirsutum*) respectively, and overexpression of *GaJAZ5* in *Arabidopsis* resulted in increased drought resistance of plants^44^. Similarly, 50, 54 and 28 *TIFY* genes were found in three cultivated cotton species (*G*. *hirsutum*, *G*. *barbadense* and *G*. *arboretum*), respectively^45^. Up to now, however, our understanding of the JAZ family members in upland cotton is still very limited. On the other hand, the recent availability of the completed genome sequence and annotation of upland cotton (*Gossypium hirsutum* L. acc. Texas Marker-1) provides us with a great opportunity to identify and characterize JAZ transcription factors in allotetraploid cotton genome^28, 29^. In this study, we identified 30 genes encoding JAZ proteins in upland cotton. Comparison of the characteristics of upland cotton JAZ family members with those of other species revealed common and diverged features of JAZ family, and may give some clues about the function of the *GhJAZ* genes. The expressions of all the *GhJAZ* genes were investigated in various tissues and different fiber developmental stages. Further study revealed that the identified GhJAZ proteins localize in cell nucleus and may form heterodimers and homodimers to perform its function in cotton fiber development.

## Results

### Identification of upland cotton *JAZ* genes

In order to globally identify the members of allotetraploid cotton JAZ family, 13 *Arabidopsis* JAZ proteins were employed as query to perform a tblastn search against upland cotton (*G*. *hirsutum* L.) acc. Texas Marker-1 (TM-1) genome in NAU-NBI and CGP-BGI databases (https://www.cottongen.org/). Totally, 30 non-redundant candidate *GhJAZ* genes were identified in upland cotton, including 15 *JAZs* from At genome and 15 *JAZs* from Dt genome (Table 1). Subsequently, with the aim to verify the reliability of the initial results, a survey was conducted to confirm the existence of the conserved ZIM domain and Jas domain with InterproScan (http://www.ebi.ac.uk/interpro/search/sequence-search). The results showed that all of the 30 putative GhJAZ proteins contain conserved ZIM and Jas domains. As upland cotton is an allotetraploid cotton species that contains At genome and Dt genome, we designated the 30 putative *JAZ* genes as *GhJAZ1-A/D* to *GhJAZ15-A/D* according to the nomenclature system applied to *Arabidopsis*, and we found only *GhJAZ10* gene pair isn’t distributed in the same At or Dt chromosome. Additionally, the length of the 30 identified GhJAZ proteins vary from 119 to 370 amino acids (Table 1).

**Table 1:** Characterization of the 30 *GhJAZ* genes identified in upland cotton genome.

Gene Name	Gene symbol	Chromosome	Location	Protein Length(aa)	Protein Mw(KD)	Protein pI
*GhJAZ1-A*	Gh_A08G2199	A08	102999359..103000765 +	252	27.36	8.53
*GhJAZ1-D*	Gh_D08G2564	D08	65322805..65324118 +	223	24.09	8.96
*GhJAZ2-A*	Gh_A06G0705	A06	19721310..19723019 +	263	28.62	8.05
*GhJAZ2-D*	Gh_D06G0810	D06	14341367..14343098 +	263	28.49	8.75
*GhJAZ3-A*	Gh_A12G2441	A12	86931181..86935899 +	370	39.03	9.71
*GhJAZ3-D*	Gh_D12G2567	D12	58573667..58577294 +	371	39.24	9.77
*GhJAZ4-A*	Gh_A05G2675	A05	44280164..44282259 +	364	39.48	9.35
*GhJAZ4-D*	Gh_D05G2975	D05	36701488..36703587 +	365	39.50	8.95
*GhJAZ5-A*	Gh_A10G2244	scaffold2455_A10	45740..47136 +	270	29.81	8.22
*GhJAZ5-D*	Gh_D10G0531	D10	5154299..5155687 +	270	29.79	8.22
*GhJAZ6-A*	Gh_A05G1155	A05	11686428..11687666 −	226	24.99	9.09
*GhJAZ6-D*	Gh_D05G1332	D05	11716272..11717510 −	226	24.88	8.37
*GhJAZ7-A*	Gh_A05G1241	A05	12558755..12559665 −	119	13.88	9.80
*GhJAZ7-D*	Gh_D05G3842	scaffold4070_D05	57087..58009 −	119	13.71	9.55
*GhJAZ8-A*	Gh_A10G0388	A10	3776510..3777261 +	120	13.81	9.69
*GhJAZ8-D*	Gh_D10G0403	D10	3648942..3649686 +	120	13.66	9.78
*GhJAZ9-A*	Gh_A01G1225	A01	68147624..68150111 +	363	38.90	8.62
*GhJAZ9-D*	Gh_D01G1406	D01	41694346..41696848 +	362	38.85	9.07
*GhJAZ10 -A*	Gh_A03G1341	A03	91837382..91839077 +	197	21.77	8.91
*GhJAZ10-D*	Gh_D02G1776	D02	60321717..60322742+	194	21.60	9.25
*GhJAZ11-A*	Gh_A07G0156	A07	1960653..1962624 +	228	24.48	5.78
*GhJAZ11-D*	Gh_D07G0152	D07	1608919..1610922 +	228	24.35	6.16
*GhJAZ12-A*	Gh_A05G0278	A05	3206768..3209220 +	216	23.15	8.06
*GhJAZ12-D*	Gh_D05G0379	D05	3151243..3153681 +	226	24.04	8.06
*GhJAZ13-A*	Gh_A05G0260	A05	2984157..2985827 +	240	25.55	9.25
*GhJAZ13-D*	Gh_D05G0352	D05	2956250..2957922 +	240	25.54	9.49
*GhJAZ14-A*	Gh_A09G0741	A09	53369098..53370369 −	125	14.36	9.83
*GhJAZ14-D*	Gh_D09G0743	D09	31625500..31626777 −	125	14.35	10.11
*GhJAZ15-A*	Gh_A01G0153	A01	1432860..1433880 +	208	23.52	10.09
*GhJAZ15-D*	Gh_D01G0196	D01	1640486..1641509 +	206	23.30	9.90

### Phylogenetic relationship of GhJAZ proteins

**Figure 1:**
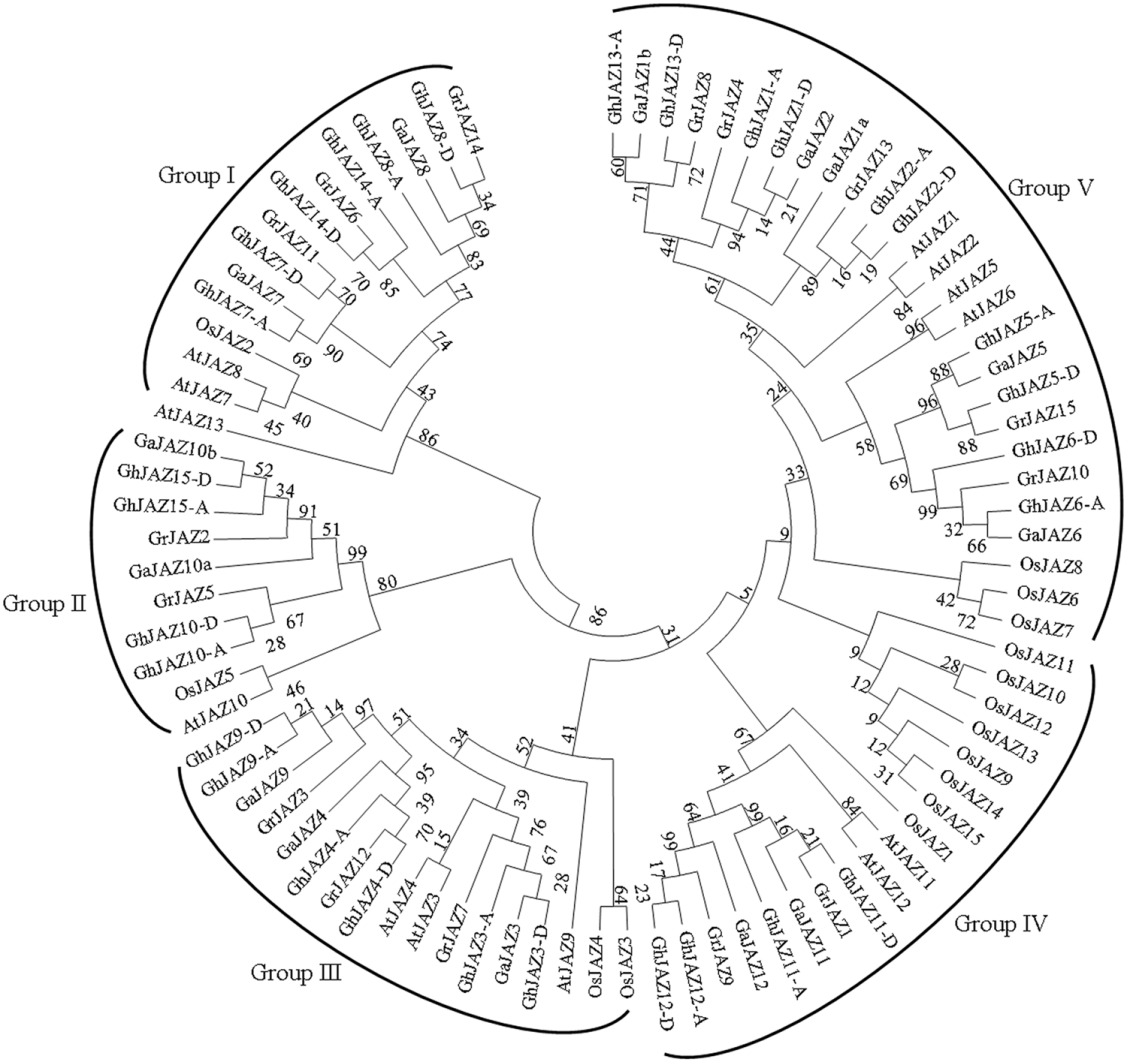
Phylogenetic relationship of JAZ proteins from upland cotton (*Gossypium hirsutum*) (Gh), *Gossypium arboreum* (Ga), *Gossypium ramondii* (Gr), *Arabidopsis thaliana* (At) and rice (*Oryza sativa*) (Os). The unrooted phylogenetic tree was constructed using MEGA 6 by Neighbor-Joining method, and the bootstrap test was performed with 1,000 iterations. The five groups are indicated with camber lines.

To reveal evolutionary relationship of JAZ transcription factor family between upland cotton (*G*. *hirsutum*, Gh), *G*. *arboreum* (Ga), *G*. *ramondii* (Gr), *Arabidopsis thaliana* (At) and rice (*Oryza sativa*, Os), an unrooted phylogenetic tree was constructed with Neiboring-Joining (NJ) method on the basis of multiple sequence alignment of 30 upland cotton (*G*. *hirsutum*) JAZ proteins, 14 *G*. *arboreum* JAZ proteins, 15 *G*. *ramondii* JAZ proteins, 15 rice JAZ proteins, and 13 *Arabidopsis* JAZ proteins. As shown in Fig. 1, the JAZ transcription factor family is divided into five groups (designated Group I to V). Among the analyzed upland cotton (*G*. *hirsutum*), *G*. *arboreum* and *G*. *ramondii* JAZ proteins, three groups (III, IV, V) of the cotton JAZs were grouped together with *Arabidopsis* JAZs rather than rice JAZs, indicating that the majority of cotton JAZs are more closely related to those of *Arabidopsis* than those of rice, which is consistent with the fact that both cotton and *Arabidopsis* are dicots and diverged more recently from a common ancestor than from the lineage leading to monocots.

### Gene structure and conserved motifs of GhJAZ proteins

**Figure 2:**
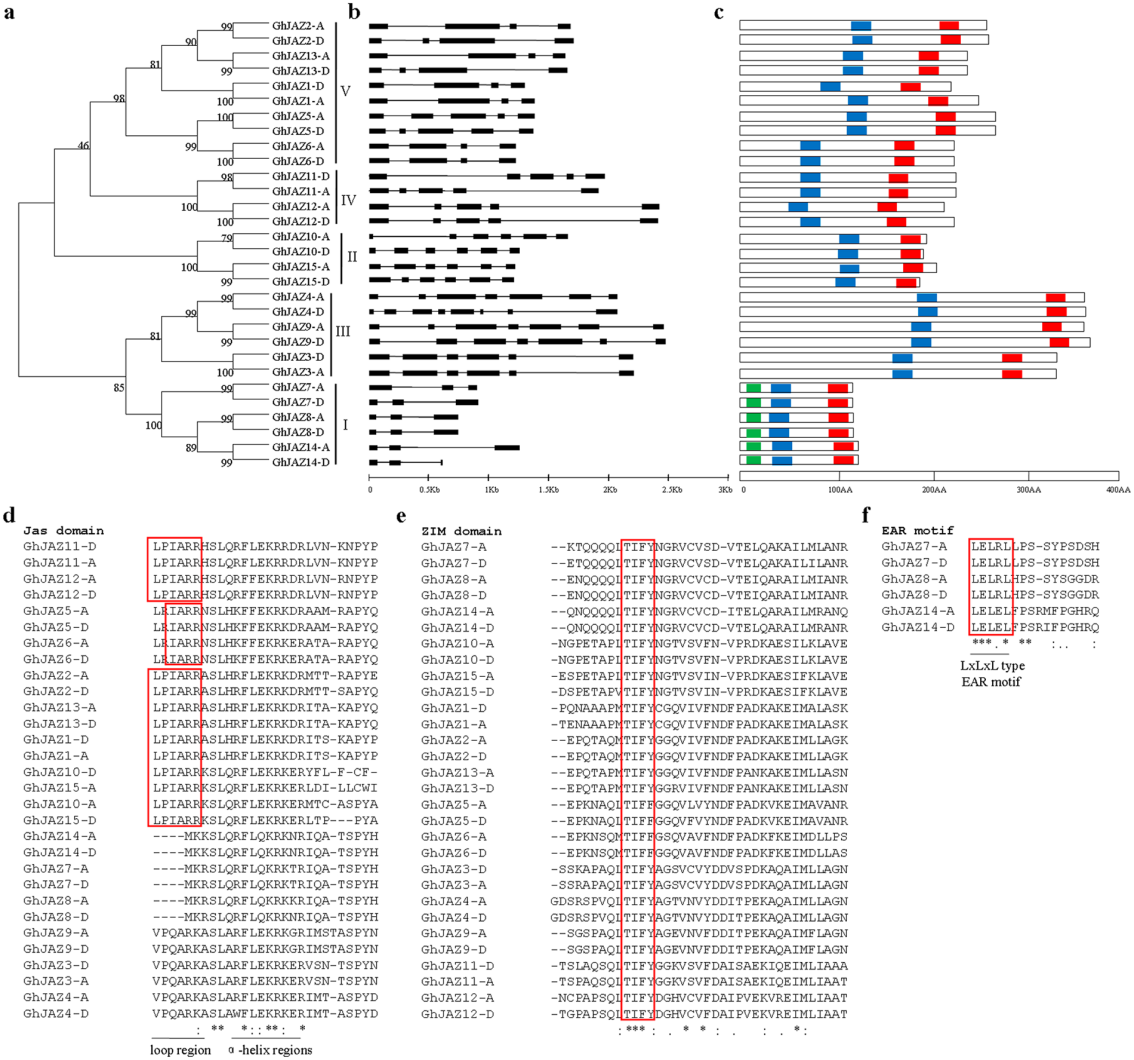
In-silico characterization of upland cotton *JAZ* genes and proteins. (**a**) The phylogenetic tree of all upland cotton JAZ proteins was constructed using Neighbor-Joining method and the bootstrap test was performed with 1,000 iterations. (**b**) The exon/intron organization of upland cotton *JAZ* genes. The boxes indicate exons and thin black lines indicate introns. (**c**) The conserved protein motifs in the upland cotton JAZ family were identified using MEME program. ZIM motif is indicated with blue, Jas motif is indicated with red and EAR motif is indicated with green. (**d**,**e**,**f**) Sequence alignment of the conserved Jas (**d**), ZIM (**e**) and EAR (**f**) motifs of upland cotton JAZ proteins by Clustal W software. The location of the three highly conserved amino acid motifs is shown.

Phylogenetic analysis was also carried out using only the amino acid sequences of the 30 GhJAZ proteins identified here. As shown in Fig. 2a, GhJAZ proteins were classified into five distinct groups (I, II, III, IV and V), whose topology was similar to that of the phylogenetic tree constructed using JAZ sequences from five plant species. With the aim to gain further insights into evolutionary relationship among *GhJAZ* genes, we investigated exon/intron structures of individual *GhJAZ* genes by alignment of cDNA sequences and corresponding genomic DNA sequences. As illustrated in Fig. 2b, all of *GhJAZ* genes contain introns, and they show great variability in exon length and intron number. Additionally, the phylogenetic tree was constructed with GhJAZ protein sequences to determine if the exon/intron organization of *GhJAZ* genes is consistent with the phylogenetic subfamilies. As expected, most *GhJAZ* genes within the same subfamily display very similar exon/intron distribution patterns in terms of exon length and intron number. For example, most *GhJAZ* genes in group I have two introns with similar possession, whereas most members within group IV contain four introns with similar possession (Fig. 2b).

Protein sequence analysis suggested that the ZIM domain in the middle of GhJAZ proteins and Jas domain in the C-terminal of GhJAZ proteins are highly conserved (Fig. 2c–e), although there are different gene lengths and great sequence diversity. As shown in Fig. 2d, α-helix regions of the Jas domain are relatively conserved, but loop regions vary in all the *JAZ* genes tested. GhJAZ1-A/D, GhJAZ2-A/D, GhJAZ10-A/D, GhJAZ11-A/D, GhJAZ12-A/D, GhJAZ13-A/D, and GhJAZ15-A/D have a short conserved LPIARR motif, which was reported to seal JA-Ile into its binding pocket at the COI1-JAZ interface. While GhJAZ5-A/D, and GhJAZ6-A/D contain the canonical C-terminal end (IARR) of the motif that contact JA-Ile but lack the N-terminal (LP) that clamp the hormone in the binding pocket, implying they may interact with GhCOI in the presence of high concentration of JA-Ile^21^. The TIFYXG motif is highly conserved in *Arabidopsis* and many other species^7, 46–48^. We found TIF(Y/F)XG motif is also highly conserved in all the 30 cotton JAZ proteins (Fig. 2e). It has been reported that the ZIM domain is required for repressor activity of several JAZ proteins, as well as formation of homodimer and heterodimer within the JAZ family, and the Jas domain is involved in a wide range of protein-protein interactions in *Arabidopsis* ^49^. Additionally, GhJAZ7-A/D, GhJAZ8-A/D and GhJAZ14-A/D lack LPIARR motif and contain an EAR motif in the N-terminal (Fig. 2c,d and f). EAR binds the co-repressor TOPLESS and represses transcriptional activation. This type of JAZ protein is unable to associate strongly with COI1 in the presence of JA-Ile and is stabilized against JA-mediated degradation.

### Expression of *GhJAZ* genes is induced by Jasmonate (JA)

**Figure 3:**
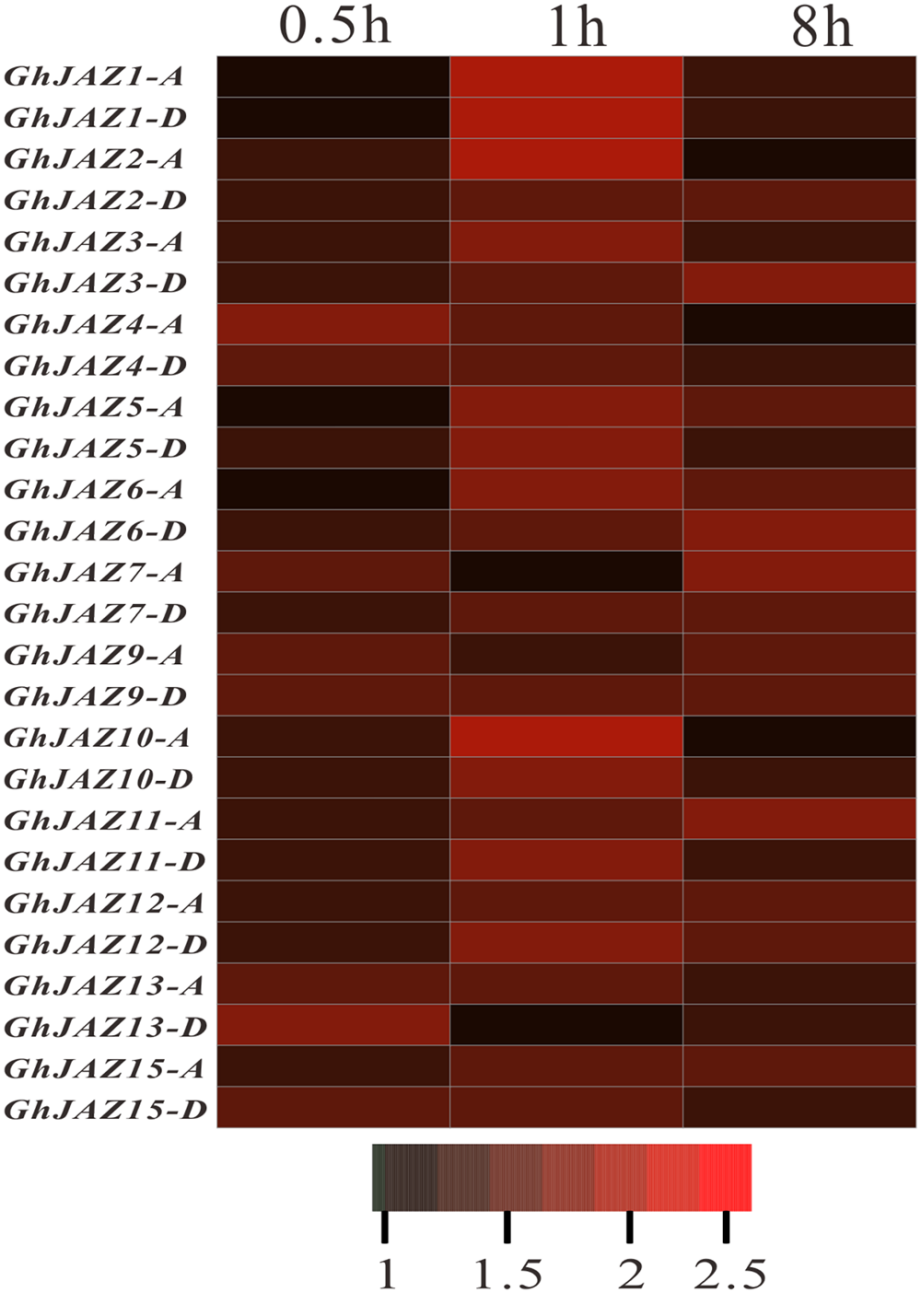
Expression profiling of *GhJAZ* genes in cotton ovules treated with jasmonate (JA). −3 DPA (three days before anthesis) ovules were cultured in a liquid BT medium supplemented with 0.5 μM JA for 0.5, 1, and 8 hours, respectively, using the same day-old ovules cultured in BT medium (without JA) as controls. Then, total RNA was isolated from the JA-treated ovules and controls, and expression of *GhJAZ* genes in the ovules was determined by real-time Quantitative RT-PCR. The relative expression value of *GhJAZ* genes was shown as ratio of gene expression levels between JA-treated ovules and controls. Data were constructed from heatmap software in the R-project. Scale, log ratio of fold change. 0.5 h, 1 h and 8 h, the ovules treated with JA for 0.5, 1 and 8 hours.

To investigate the Jasmonate(JA)-induced expression of *GhJAZ* genes, ovules at −3 DPA (3 days before anthesis) were cultured in a liquid medium supplemented with or without 0.5 μM JA *in vitro* (see Methods). After 0.5 hour of culture, we found that expression levels of the *GhJAZ* genes in the JA-treated ovules were remarkably increased, relative to those controls. Most of the *GhJAZ* genes were strongly induced by JA, and their expression levels in the JA-treated ovules were 2- − 2.5-fold (or more) higher than those in the controls after 1 hour of culture. With the prolonged JA treatment, however, the induced expression of these *JAZ* genes was gradually declined, although expression levels of these genes in the JA-treated ovules were still higher than those in the control ovules (Fig. 3). These results indicated that the most *GhJAZ* genes could be induced in cotton ovules by JA, implying they may participate in JA signaling pathway during ovule and fiber development of cotton.

### Expression patterns of *GhJAZ* genes in cotton tissues

**Figure 4:**
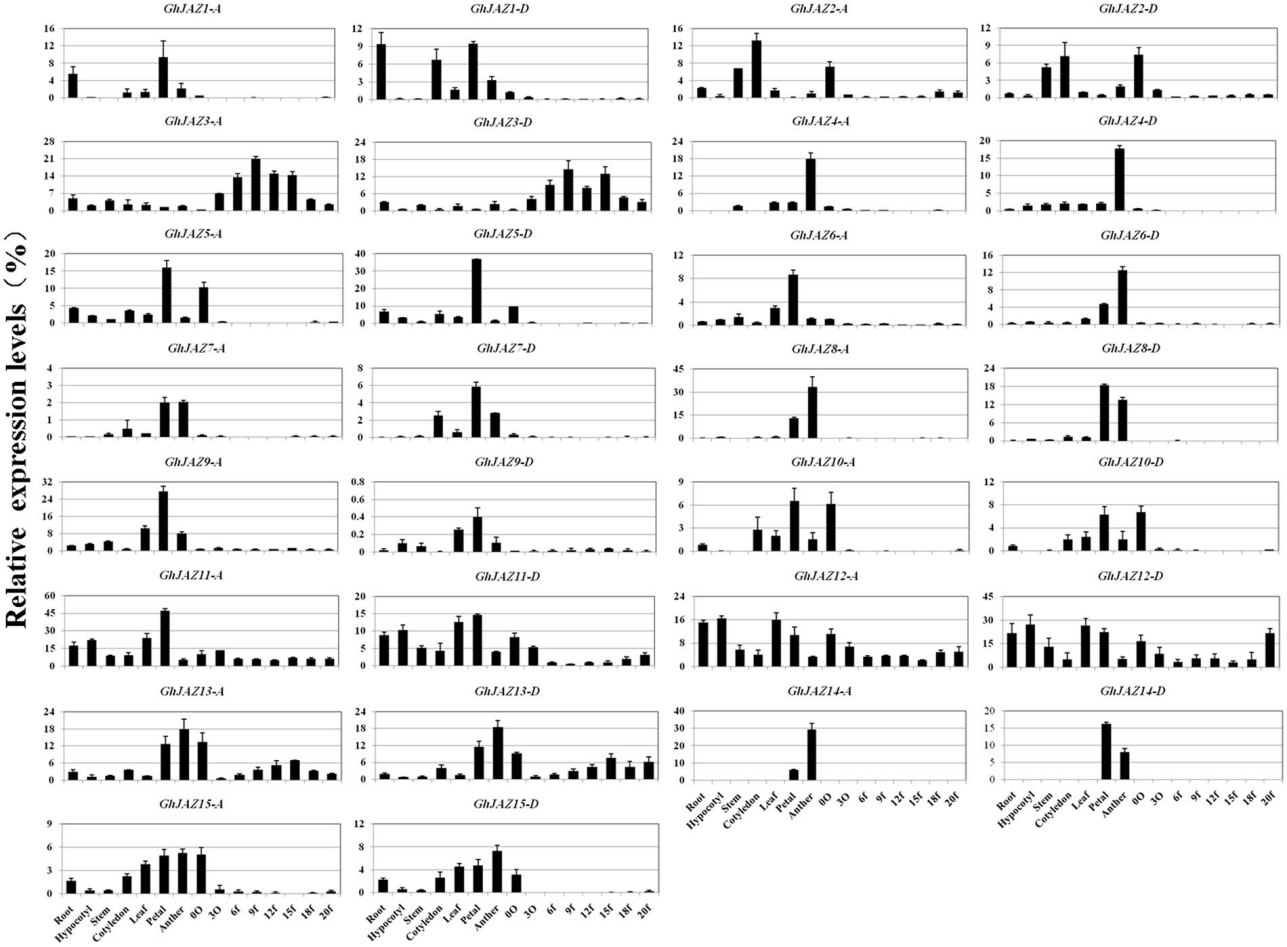
Quantitative RT-PCR analysis of expression of *GhJAZ* genes in upland cotton tissues. Total RNAs were isolated from different cotton tissues. Relative value of expression of *GhJAZ* genes in cotton tissues was shown as percentage of *GhUBI1* expression activity. Data presented in the quantitative RT-PCR analysis are mean values and standard deviation of three biological replicates of plant materials and three technical replicates in each biological sample. 0 O and 3 O, 0 and 3 DPA (day post anthesis) ovules with fibers; 6 f–20 f, 6 to 20 DPA fibers.

To predict possible functions as well as to identify probable functional redundancy through overlapping expression patterns for the cotton *JAZ* genes, we determined expression levels of all 30 genes by real-time quantitative RT-PCR. We used mRNA isolated from cotton roots, hypocotyls, stems, leaves, cotyledons, petals, anthers, ovules at 0 DPA (days post anthesis), ovules and fibers at 3 DPA, and fibers at 6–20 DPA. All expression levels, relative to expression of a cotton polyubiquitin gene (*GhUBI1*), are shown in numerical order in Fig. 4. The results indicated that the expression patterns are similar among the same JAZ group. For example, Group I *JAZs* (*GhJAZ7/8/14-A/D*) were preferential or specific expressed in anthers or petals. Group II *JAZs* (*GhJAZ10/15-A/D*) were mainly expressed in cotyledons, leaves, petals, anthers and 0 DPA ovules. All Group III genes (*GhJAZ3/4/9-A/D*) were tissues-specifically expressed genes. *GhJAZ3-A/D* were preferentially expressed in elongating fibers (6–20 DPA cotton fibers), *GhJAZ4-A/D* were specifically expressed in anther, and *GhJAZ9-A/D* were specifically expressed in petals. Four members in Group IV (*GhJAZ11-A/D* and *GhJAZ12-A/D*) were ubiquitously expressed in all cotton organs/tissues, and showed relatively high expression level during cotton fiber development, especially during fiber initiation. Group V, which is composed of 10 members (*GhJAZ1/2/5/6/13-A/D*), seems to be widely expressed in various tissues, including roots, leaves, petals, anthers and early stages of developing ovules and fibers (Fig. 4).

The experimental results also revealed that 14 *GhJAZs* (*GhJAZ2-A/D*, *GhJAZ5-A/D*, *GhJAZ10-A/D*,, *GhJAZ11-A/D*, *GhJAZ12-A/D*, *GhJAZ13-A/D* and *GhJAZ15-A/D*) were highly expressed in initiating fiber cells (0 DPA ovules) of cotton. Eight genes (including *GhJAZ3-A/D*, *GhJAZ11-A/D*, *GhJAZ12-A/D* and *GhJAZ13-A/D*) were expressed at relatively higher levels in elongating cotton fibers (3–20 DPA). Twelve genes (*GhJAZ4-A/D*, *GhJAZ7-A/D*, *GhJAZ8-A/D*, *GhJAZ13-A/D*, *GhJAZ14-A/D* and *GhJAZ15-A/D*) had highly accumulating transcripts in anthers (Fig. 4). The above data suggested that different members in *GhJAZ* family may participate in different tissue development, and some *GhJAZs* may function in cotton fiber development.

### Comparison of expression levels of *GhJAZ* genes between Xuzhou142 and its fiberless mutant (*fl*)

**Figure 5:**
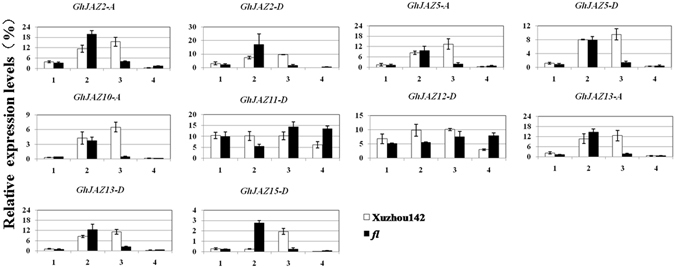
Expression profiling of *GhJAZ* genes in early developing ovules and fibers of wild type cotton (Xuzhou142) and its fiberless mutant (*fl*). Total RNAs were isolated from different development stage ovules. Relative value of the expression of *GhJAZ* genes in ovules was shown as percentage of *GhUBI1* expression activity. 1, −2DPA ovule; 2, −1DPA ovule; 3, 0DPA ovule; 4, 1DPA ovule. Data presented in the quantitative RT-PCR analysis are mean values and standard deviation of three biological replicates of plant materials and three technical replicates in each biological sample. DPA, day post (after) anthesis.

To further investigate the potential function of *GhJAZ* genes in cotton fiber initiation, we chose 10 *GhJAZ* genes, which showed relatively high expression in 0 DPA ovules, to test the transcript level difference between cotton cultivar Xuzhou142 (wild type) and its fiberless mutant (*fl*) in 0 DPA ovules with initiating fiber cells. As shown in Fig. 5, *GhJAZ2-A/D*, *GhJAZ5-A/D*, *GhJAZ10-A*, *GhJAZ13-A/D* and *GhJAZ15-D* showed the similar variation trend in Xuzhou142 and *fl* ovules. The relative expression levels of these genes were increased from −2 DPA ovules and reached to a peak in 0 DPA ovules, and then decreased to a very low level in 1 DPA Xuzhou142 ovules. Copmared with Xuzhou142, the expression patterns of these genes were very different in *fl* mutant. Their mRNAs were accumulated in −1 DPA ovules but these genes showed no or very weak expression in −2, 0 and 1 DPA ovules. *GhJAZ11-D* and *GhJAZ12-D* showed similar patterns and kept relatively high levels during cotton fiber initiation between Xuzhou142 and *fl*. The differential expression between Xuzhou142 and its mutant *fl* implied that *GhJAZ* genes may participate in fiber initiation of cotton.

### Subcellular localization of GhJAZ proteins

**Figure 6:**
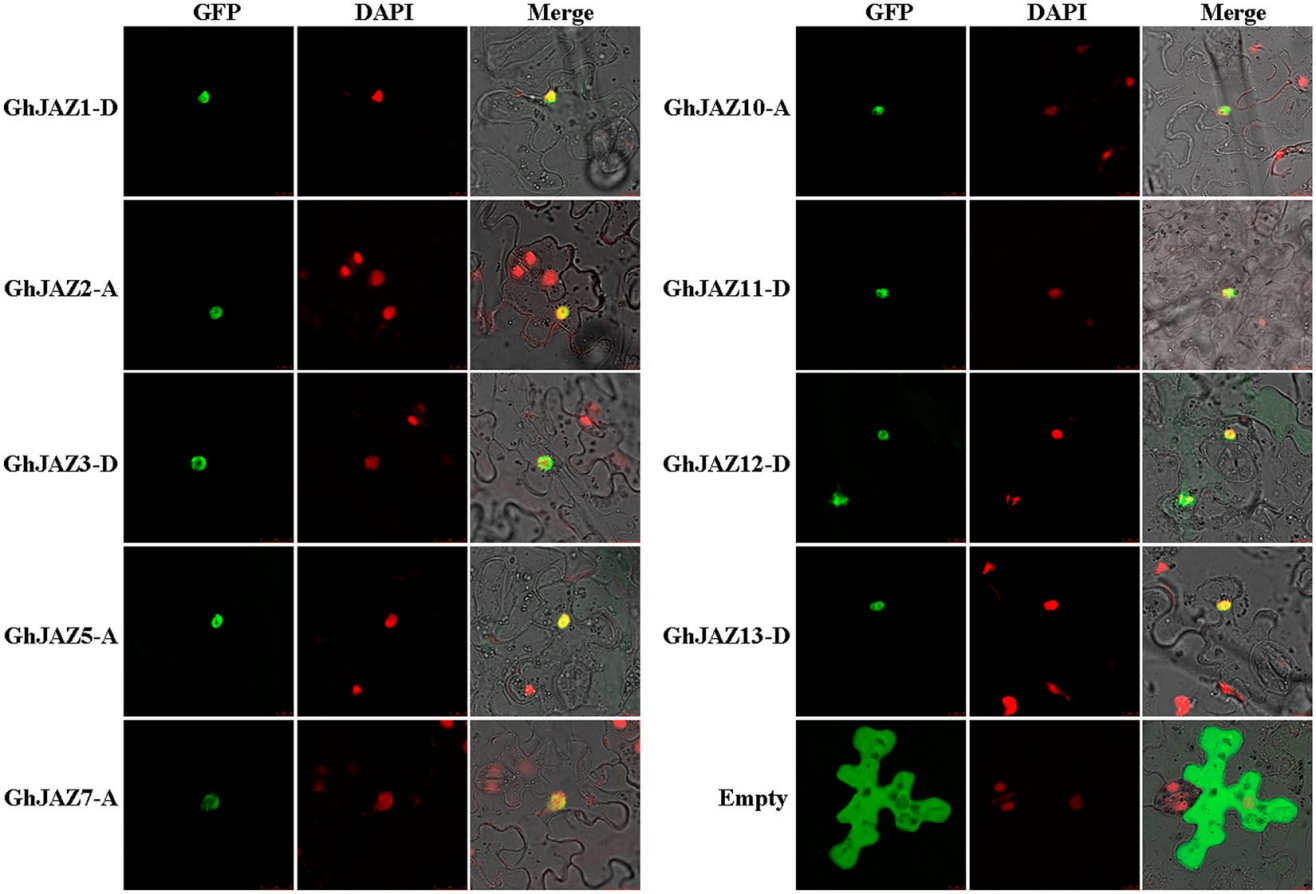
Assay of subcellular localization of GhJAZ proteins in leaf cells of tobacco (*Nicotiana benthamiana*). The GhJAZ::eGFP constructs were transferred into tobacco leaf cells, respectively. Then, the transformed leaves were stained by DAPI (a nuclear-specific dye). Fluorescence images were obtained using confocal microscopy. Merge means image GFP and image DAPI merged with its bright-field photograph in the same cell. Scale bar = 50 µm.

To assay subcellular localization of the GhJAZ proteins, we chose 9 of 30 *GhJAZ* genes, which are strongly expressed in the very young ovules during cotton fiber initiation, to construct the *GhJAZs::eGFP* fusion vectors under control of *CaMV* 35 S promoter, and transiently expressed these fusion proteins in leaves of tobacco (*Nicotiana benthamiana*). The eGFP fluorescence in leaves was observed under a Leica Confocal laser scanning microscopy. As shown in Fig. 6, green GFP fluorescence was mainly accumulated in nuclei of leaf epidermal cells, indicating that the GhJAZ proteins localized in the cell nucleus.

### Interaction of GhJAZ proteins

**Figure 7:**
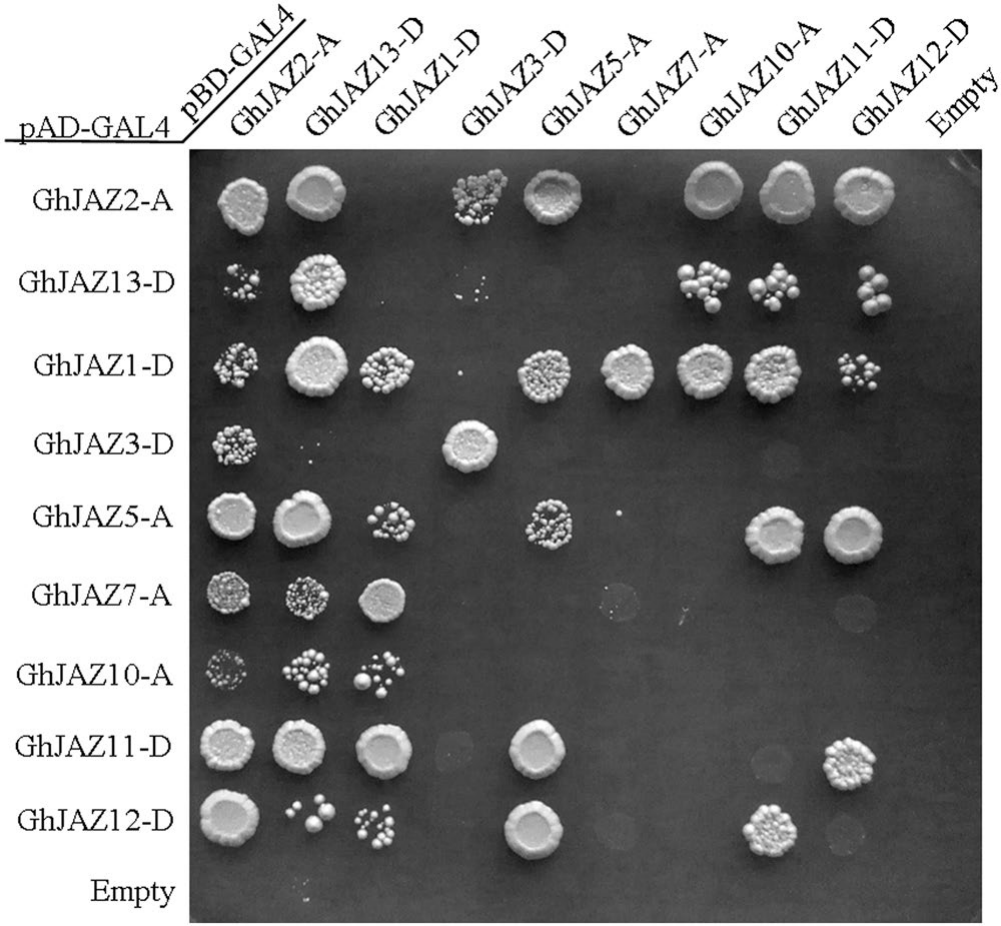
Yeast two-hybrid assay of interactions among GhJAZ proteins. The coding sequences of the *GhJAZ* genes were cloned into the yeast two-hybrid vectors pGADT7 and pGBKT7, and introduced into yeast cells. Interactions among the GhJAZ proteins were analyzed by yeast mating. Positive transformants were determined on QDO (SD-Trp/-Leu/-Ade/-His) nutritional selection medium.

Recently, two labs reported JAZ proteins could interact with each other and function as homodimer and heterodimer in *Arabidopsis* ^50, 51^. To understand whether and how cotton JAZ proteins interact with each other, yeast two-hybrid technology was employed to analyze the interaction among GhJAZ proteins. We chose nine GhJAZ proteins as candidates to elucidate the interaction of GhJAZs. All 9 GhJAZs showed no selfactivation of transcription. As shown in Fig. 7, GhJAZ1-D, GhJAZ2-A, GhJAZ5-A and GhJAZ13-D (belonged to group V), and GhJAZ3-D (belonged to group III) could interact with itself as homodimers, respectively, while the remaining GhJAZ proteins can not form homodimers. Furthermore, GhJAZ1-D, GhJAZ2-A, GhJAZ5-A and GhJAZ13-D, which belong to group V, could interact with each other and also formed heterodimers with other cotton JAZ proteins. Three proteins (GhJAZ10-A, GhJAZ11-D and GhJAZ12-D) from group II and IV could interact with nearly all of the group V proteins, while they also interacted with GhJAZ11-D and GhJAZ12-D, but showed only weak interaction signals with GhJAZ3-D (group III) and GhJAZ7-A (group I). These widely interaction between GhJAZ proteins suggested that each of the cotton JAZ proteins may display isoform selectivity in formation of heterodimers and homodimers in cells during cotton development.

### GhJAZ proteins interact with GhCOI1, GhMYC2/3 and GhNINJA

**Figure 8:**
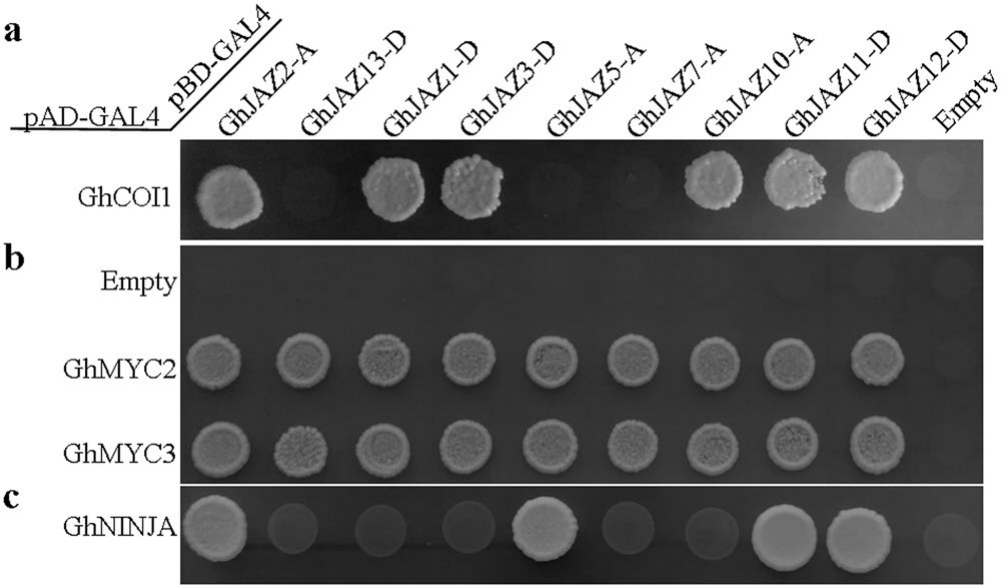
Yeast two-hybrid assay of interactions among GhJAZ proteins with GhCOI1, GhMYC2/3 and GhNINJA. The coding sequences of the *GhCOI1*, *GhMYC2/3* and *GhNINJA* genes were cloned into the yeast two-hybrid vector pGADT7, and the coding sequences of the *GhJAZ* genes were cloned into the yeast two-hybrid vector pGBKT7. The constructs were introduced into yeast cells, respectively. Interactions among the GhJAZ proteins and GhCOI1, GhMYC2/3, GhNINJA proteins were analyzed by yeast mating. (**a**) Transformants related to GhCOI1 grew on QDO (SD-Trp/-Leu/-Ade/-His) nutritional selection medium with 20 µM COR. (**b,c**) Transformants related to GhMYC2/3 and GhNINJA grew on QDO nutritional selection medium.

Chini and coworkers reported COI1/JAZs/MYC2 as the core jasmonic acid signalling module in *Arabidopsis* ^14, 52^. NINJA connects the JAZ proteins with the TPL co-repressors and function as negative regulators of jasmonate responses^20^. JA-Ile and the phytotoxin coronatine (COR) directly induce the interaction between the receptor AtCOI1 and several AtJAZ proteins at physiological concentrations^12, 19, 53^. To investigate similar interactions in cotton, we performed yeast two-hybrid assay for the cotton homologs, using empty vectors containing the activation domain or binding domain as negative controls. The experimental results showed that GhJAZ1-D, GhJAZ2-A, GhJAZ3-D, GhJAZ10-A, GhJAZ11-D and GhJAZ12-D interacted with GhCOI1 in the presence of 20 µM COR (Fig. 8a). All of these 9 GhJAZ proteins interacted with both GhMYC2 and GhMYC3 (Fig. 8b). GhNINJA could interact with GhJAZ2-A, GhJAZ5-A, GhJAZ11-D and GhJAZ12-D (Fig. 8c). These results imply that GhJAZ proteins may recruit GhNINJA, and function as repressors of JA signaling pathway by interacting with GhMYC2 and GhMYC3, and they may be targeted for proteasome degradation by SCF^COI1^ and activating the JA responses by releasing GhMYC2 and GhMYC3.

### Interaction partners of GhJAZ proteins with cotton fiber initiation factors

**Figure 9:**
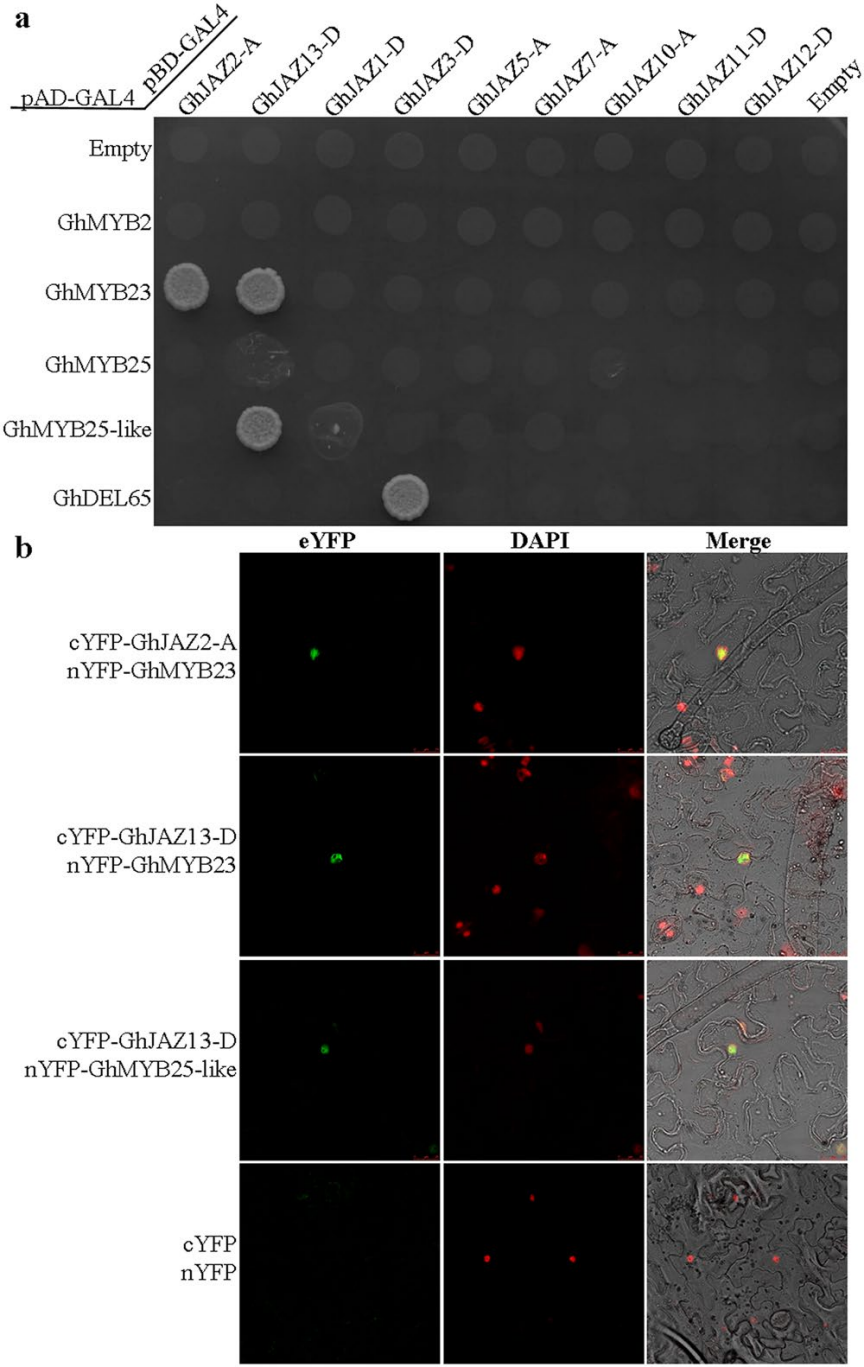
Interactions among GhJAZ proteins and cotton fiber initiation factors. (**a**) Yeast two-hybrid assay of GhJAZ proteins interacted with cotton fiber initiation factors. The coding sequences of the *GhMYB2*, *GhMYB23*, *GhMYB25*, *GhMYB25-like* and *GhDEL65* genes were cloned into the yeast two-hybrid vectors pGADT7, and the coding sequences of the *GhJAZ* genes were cloned into the yeast two-hybrid vector pGBKT7. The constructs were introduced into yeast cells, respectively. Interactions among the GhJAZs and the GhMYB2, GhMYB23, GhMYB25, GhMYB25-like, GhDEL65 proteins were analyzed by yeast mating. Transformants grew on QDO (SD-Trp/-Leu/-Ade/-His) nutritional selection medium. (**b**) Bimolecular fluorescence complementation (BiFC) assay of the interactions of GhJAZ2-A, GhJAZ3-D or GhJAZ13-D (fused with C-terminal fragment of YFP) with GhMYB23 or GhMYB25-like (fused with N-terminal fragment of YFP). Construct pairs indicated on the left were coexpressed in leaves of tobacco (*Nicotiana benthamiana*) (see Methods). YFP fluorescence was detected in epidermal cells of tobacco leaves. The cell nuclei are indicated by DAPI staining.

In *Arabidopsis*, JAZ interact with GL3 and GL1, which are essential components of WD-repeat/bHLH/MYB complex, to regulate JA-mediated trichome initiation in *Arabidopsis* ^39^. In cotton, GhMYB25-like functions as upstream of GhMYB25 to regulate cotton fiber initiation^34, 42^, and GhJAZ2 could interact with GhMYB25-like to repress cotton fiber initiation^35^. To further investigate whether GhJAZs could interact with the key transcription factors to control fiber development, we used yeast two-hybrid assay to explore interactions of the 9 GhJAZ proteins with major members of cotton fiber initiation factors, including GhMYB2, GhMYB23 (GL1 homolog), GhMYB25 and GhMYB25-like, and GhDEL65 (GL3 homolog). As shown in Fig. 9a, we only detected the interaction between GhJAZ2-A and GhMYB23, GhJAZ13-D and GhMYB23/25-like, and GhJAZ3-D and GhDEL65, respectively. Then, bimolecular fluorescence complementation (BiFC) assay was employed to verify these interactions in tobacco (*Nicotiana benthamiana*). The C-terminal fragment of yellow fluorescent protein (cYFP) was respectively ligated with GhJAZ2-A, GhJAZ13-D and GhJAZ3-D, while GhMYB23, GhMYB25-like and GhDEL65 were individually fused with the N-terminal fragment of YFP (nYFP). The experimental results demonstrated that coexpression of cYFP-GhJAZ2-A/nYFP-GhMYB23, cYFP-GhJAZ13/nYFP-GhMYB23, and cYFP-GhJAZ13/nYFP-GhMYB25-like resulted in strong YFP fluorescence in the nuclei of tobacco leaf epidermal cells, but the interaction between cYFP-GhJAZ3-D and nYFP-GhDEL65 was not detected *in vivo* by BiFC (Fig. 9b). These results suggest that molecular mechanism of regulating cotton fiber cell differentiation may be similar to that of *Arabidopsis* leaf trichomes/root hairs differentiation. GhJAZ proteins may participate in cotton fiber differentiation by interacting with fiber initiation factors, such as GhMYB23 and GhMYB25-like.

## Discussion

Jasmonate ZIM domain (JAZ) proteins are plant-specific proteins and act as transcriptional repressors of JA-responsive genes. JA regulates many aspects of plant growth, development, and defense^3–6^. JAZ family proteins, as the important transcription factors, have been identified in some plant species, like rice^46^, Chickpea^54^, grape^55^, soybean^47^, rubber tree^48^, *Salvia miltiorrhiza* ^56^, *G*. *ramondii* ^43^ and maize^57^. Until more recently, Zhao *et al*. reported 25 *JAZ* genes in upland cotton and only analyzed their phylogenetic relationship and exon-intron structure^44^. Also, Sun and colleagues (2017) identified 24 *JAZ* genes in upland cotton, and only reported some of *GhJAZ* genes responding to plant hormones JA, GA, or ABA^45^. In this study, we identified a total of 30 *GhJAZ* genes, which consist of 15 gene pairs in At and Dt genome of upland cotton (Table 1), and provided more detailed data of this JAZ family, especially involving in fiber development of cotton. The identified GhJAZ family proteins contain conserved ZIM and Jas domains, and can be divided into five groups (I to V). The members in different groups of GhJAZ family show great divergence in sequence length and exon-intron organization, but those in the same group were closely related in these features (Figs 1 and 2).

A recent study reported 4778 and 2548 TFs genes existed in upland cotton (*G*. *hirsutum*) genome and diploid cotton (*G*. *raimondii*) genome, respectively^29^. According to the family assignment rules, 1717 loci TFs are identified and classified into 58 families in TAIR10 (http://planttfdb.cbi.pku.edu.cn/index.php?sp=Ath). That means there are about 1.4 and 1.5 fold duplication of TF genes in upland cotton (At or Dt genome) and *G*. *raimondii* (D genome) compared with TF genes in *Arabidopsis*. Thus, the gene duplicates of *JAZ* family are less than those of the other TF gene families in upland cotton. Compared with the *JAZ* gene numbers in diploid plant species (15 in *G*. *ramondii*, 14 in *G*. *arboreum*, 13 in *Arabidopsis*, 15 in rice, 11 in grape, and 12 predicted *JAZ* genes in *H*. *brasiliensis*), on the other hand, we found the number of *JAZ* genes is closely in all plant species, implying nearly no *JAZ* duplication has occurred in these species during evolution.

Previous study reported that continuous exogenous JA application inhibited fiber elongation of cotton *in vitro* and *in vivo*, indicating the negative relationship of JA and fiber elongation^58^. Also, JA-associated metabolism is related to cotton fiber initiation^59^. In our work, we found that most of the *GhJAZ* genes could be induced by JA in the ovules during fiber initiation. Furthermore, *GhJAZ3-A/D* was preferential expressed in cotton fiber elongation and secondary cell wall formation, *GhJAZ2-A/D*, *GhJAZ5-A/D*, *GhJAZ10-A-D*, *GhJAZ13-A/D and GhJAZ15-A/D* showed relatively high expression during cotton fiber initiation, while *GhJAZ2-A/D*, *GhJAZ11-A/D*, *GhJAZ12-A/D* and *GhJAZ13-A/D* were ubiquitously high expressed during cotton fiber development. These results implied that these *JAZ* genes may be important for cotton fiber initiation and elongation.

Comparative analysis of gene expression patterns in mutant and wild type provides a powerful approach for investigating genes involved in key stages of cotton fiber development. Previous study reported JA-associated biosynthesis genes (eg: *AOCs*) are preferentially expressed in −1 DPA cotton ovules, and their expression levels in *fl* mutant were generally higher than those in Xuzhou142. Parallel up-regulation of expression of *AOCs* may be important for normal fiber initiation, while overproduction of *AOCs* might disrupt normal fiber development^59^. In this study, wild type Xuzhou142 and its fiberless mutant *fl* with a genetic locus difference were used to identify differentially expressed *GhJAZ* genes in fibers. We found the transcripts of *GhJAZ2-A/D*, *GhJAZ5-A/D*, *GhJAZ10-A*, *GhJAZ13-A* and *GhJAZ15-D* were largely accumulated in 0 DPA Xuzhou142 ovules and then decreased to very low levels, while expression levels of these genes in *fl* mutant advanced the peaking time to −1 DPA and nearly none expression of them was detected during the other three phases (Fig. 5). These data suggested that keep high expression of these *JAZ* genes in ovules until 0 DPA may promote fiber initiation of cotton.

Additionally, yeast two-hybrid assay revealed that GhJAZ1-D, GhJAZ2-A, GhJAZ5-A, GhJAZ11-D, GhJAZ12-D and GhJAZ13-D could interact widely with other cotton JAZ proteins, while GhJAZ3-D and GhJAZ7-A only interacted with several GhJAZ proteins, implying that the GhJAZ proteins may display isoform selectivity in formation of heterodimers and homodimers. Most of GhJAZs could interact with the core jasmonic acid signalling proteins, GhCOI1, GhMYC2, GhMYC3 and GhNINJA (Fig. 8), suggesting they may recruit GhNINJA, and function as repressors of JA signaling pathway by interacting with GhMYC2 and GhMYC3. It has been supposed that mechanism of regulating fiber cell differentiation of cotton may be similar to that of leaf trichomes/root hairs differentiation of *Arabidopsis*, and TFs also play important roles in regulating fiber initiation and elongation^30–36^. In this work, we found GhJAZ2-A and GhJAZ13-D could interact with GhMYB23, which are homologs of the essential components of WD-repeat/bHLH/MYB complex, suggesting GhJAZ proteins associated-regulating cotton fiber initiation may be dependent on GhMYB23. Meanwhile, we found GhJAZ13-D could interact with GhMYB25-like. Similarly, a previous study revealed that GhJAZ2 interacts with GhMYB25-like^35^. As originated from Dt genome of cotton, furthermore, the GhJAZ2 is renamed as GhJAZ2-D in present study, and shares 69% identity with GhJAZ13-D. *GhMYB25-like* is a fiber specific gene and maximal expression occurred in ovules at −1 to +3 DPA. GhMYB25-like is a key regulatory component, acting upstream of GhMYB25, in the pathway that specifically regulates epidermal cell differentiation to form cotton seed trichomes (i.e. fibers)^34^. Thus, these findings suggest that GhJAZ proteins may regulate fiber differentiation and development by interacting with cotton fiber initiation factors.

## Methods

### Plant Growth Conditions

Upland cotton (*Gossypium hirsutum* cv. Coker312, Xuzhou142 and its fiberless mutant *fl*)^34^ seeds were surface sterilized with 70% (v/v) ethanol for 1 min and 10% hydrogen peroxide for 2 h, followed by washing with sterile water. The sterilized seeds were germinated and grew on one-half strength Murashige and Skoog (MS) medium (12-h-light/12-h-dark cycle, 28 °C), and the seedlings were transplanted into soil for further growth to maturation. Tissues for DNA and RNA extraction were collected from cotton plants.

### Identification of *GhJAZ* genes

We identified *JAZ* genes from upland cotton genome sequences in the COTTONGEN (https://www.cottongen.org/)^29^. The *JAZ* genomic sequences and CDS sequences extracted from COTTON GENOME PROJECT (CGP, http://cgp.genomics.org.cn/page/species/index.jsp)^28^ were compared to infer the exon/intron organization of *JAZ* genes. And the *GhJAZ* cDNAs were cloned using the total RNA as templates from different tissues of upland cotton and gene-specific primers (Supplementary Table 1).

### DNA and protein sequence analysis and conserved motif identification

DNA and protein sequences were analyzed using DNASTAR software (DNAStar, MD, USA). Protein domains and significant sites were identified using Motif Scan (http://myhits.isb-sib.ch/cgi-bin/motif_scan) and InterproScan (http://www.ebi.ac.uk/interpro/search/sequence-search). Sequence alignment of Jas and ZIM domain was performed using ClustalW (http://www.ebi.ac.uk). Phylogenetic analysis was performed to determine evolutionary relationships among protein sequences. A minimum evolution tree was generated using MEGA6 (http://www.megasoftware.net/). A bootstrap analysis with 1000 replicates was performed to assess the statistical reliability of the tree topology.

### Quantitative RT-PCR analysis

Total RNA was extracted from roots, hypocotyls, cotyledons, leaves, petals, anthers, ovules, and developing ovules and fibers (0–20 DPA, days post anthesis). RNA was purified using the Qiagen RNeasy kit according to the manufacturer’s instructions. Firststrand synthesis of cDNA was performed using Moloney murine leukemia virus reverse transcriptase (Promega) according to the manufacturer’s instructions. Expression of cotton genes in different tissues and developmental fibers was analyzed by real-time quantitative RT-PCR using the fluorescent intercalating dye SYBR Green in the detection system (MJ Research; Option 2). A cotton polyubiquitin gene (*GhUBI1*, GenBank accession no. EU604080) was used as a standard control in the RT-PCR. A three-steps RT-PCR procedure was performed in all experiments using a method described earlier^60, 61^. In brief, cDNAs reverse-transcribed from total RNA was used as templates in real-time PCR with gene-specific primers (Supplementary Table 2). PCR was performed using SYBR Green Real-Time PCR Master Mix (Toyobo) according to the manufacturer’s instructions. The relative expression of target genes was determined using the comparative cycle threshold method. To achieve optimal amplification, PCR conditions for every primer combination were optimized for annealing temperature and Mg^2+^ concentration. PCR products were confirmed on an agarose gel.

### *In vitro* ovule culture of cotton and analysis of *GhJAZ* gene expression

−3 DPA (three days before anthesis) ovules were harvested from cotton plants growing in soil at 8 to 9 o’clock in the morning. *In vitro* ovule culture of cotton was performed by the method described previously^62^. Cotton ovules was cultured in liquid BT medium without (control) or with 0.5 μM jasmonate (JA). After 0.5, 1 and 8 hours, the cultured ovules were collected for RNA isolation, respectively. The expression levels of *GhJAZ* genes were analyzed by real-time quantitative RT-PCR described as above. The relative expression value of *GhJAZ* genes was shown as ratio of gene expression levels between JA-treated ovules and controls (without JA treatment). Data were constructed from heatmap software in the R-project.Scale, log ratio of fold change (https://www.r-project.org/). More than three groups were tested in each replicate.

### Yeast two-hybrid assay

For directed yeast two-hybrid assays of protein–protein interaction between GhJAZ proteins, the coding sequences of *GhJAZ* genes amplified by PCR using Pfu DNA polymerase and gene-specific primers (Supplementary Table 1) were cloned into the yeast two-hybrid vectors pGBKT7 (bait vector) and pGADT7 (prey vector), creating fusions to the binding domain and activation domain of the yeast transcriptional activator GAL4, respectively. The bait vector and prey vector were transformed into Y187 and AH109, respectively. The yeast two-hybrid was obtained by mating Y187 and AH109^63^. The transformants were further streaked on quadruple dropout medium (QDO medium, SD/–Trp/–Leu/–His/–Ade)^64^.

### Assay of bimolecular fluorescence complementation (BiFC)

The coding sequences of *GhJAZ2-A*, *GhJAZ3-D* and *GhJAZ13-D* were fused with the C-terminal fragment of YFP (cYFP) to produce pSAT1-cYFP-GhJAZ constructs, and the coding sequences of *GhMYB23*, *GhMYB25-like* and *GhDEL65* were fused with the N-terminal fragment of YFP (nYFP) to generate pSAT1-nYFP-MYB23/-MYB25-like/-DEL65 vectors, respectively. The constructs were transferred into *Agrobacterium tumefaciens* (strain GV3101) by electroporation. two types of the transformed *Agrobacterium* cells (each with pSAT1-cYFP-GhJAZ construct, and another with pSAT1-nYFP-MYB23 or -MYB25-like or -DEL65 vector) were cosuspended in 10 mM MES buffer (pH 5.7) with 10 mM MgCl_2_ and 0.6 mM acetosyringone, and coinfiltrated into abaxial sides of tobacco (*Nicotiana benthamiana*) leaves with a needleless syringe. The tobacco plants were placed for 48 hours, and all construct pairs were transiently coexpressed in tobacco leaves, respectively. Then, YFP fluorescence in epidermal cells of tobacco leaves was detected under a Leica TCS SP5 confocal laser scanning microscope (Leica Co., Germany).

## Electronic supplementary material

Supplementary tables 1 and 2
