# Supplementary material for: Genome-wide identification and characterization of JAZ gene family in upland cotton (Gossypium hirsutum)
Source: Sci Rep. 2017 Jun 5;7:2788. doi: 10.1038/s41598-017-03155-4 (PMC5459830; doi:10.1038/s41598-017-03155-4)
Supplement: Supplementary file 1 — Supplementary tables 1 and 2 [file 41598_2017_3155_MOESM1_ESM.pdf]

Genome-wide identification and characterization of *JAZ* gene family in upland cotton (*Gossypium hirsutum*)

Wen Li, Xiao-Cong Xia, Li-Hong Han, Ping Ni, Jing-Qiu Yan, Miao Tao, Geng-Qing Huang, Xue-Bao Li

Supplementary Information

Supplementary Table 1. Gene-specific primers used in isolation of *GhJAZ* genes and vector construction.

Primer name	Primer sequence
JAZ1-D P1	5'-CTTCATATGATGTTTGGTTCACCGGAGAAAC-3'
JAZ1-D P2	5'-CTTGGATCCCTACTGCAGTGATTCAACAGCT-3'
JAZ2-A P1	5'-CTTCATATGATGAATATGTCGTGTTCACCGGA-3'
JAZ2-A P2	5'-CTTGGATCCCTACGGAGATTGAGCAGCCAAACC-3'
JAZ3-D P1	5'-CTTCATATGATGGAGAGAGATTTTCTCGGTT-3'
JAZ3-D P2	5'-CTTGGATCCTTAATTGATGGCTGTAAAAGGAC-3'
JAZ5-A P1	5'-CTTCATATGATGTCTAATTTAGGGCAAAAATC-3'
JAZ5-A P2	5'-CTTGGATCCCTATAAGTTGAGATCAATGTC-3'
JAZ7-A P1	5'-CTTCATATGATGAGACGAAACTGCAACTTGG-3'
JAZ7-A P2	5'-CTTGGATCCTTAATGGTAAGGAGAGGTAGC-3'
JAZ10-A P1	5'-CTTGAATTCATGTCGAGAGCTAGCGTCGAGCT-3'
JAZ10-A P2	5'-CTTGGATCCCTAGCAAGCATATGGAGATGCAC-3'
JAZ11-D P1	5'-CTTCATATGATGGAAGAAGAAGCTGAATCAC-3'
JAZ11-D P2	5'-CTTGGATCCTTACGCAACATGAGCTGGAGCT-3'
JAZ12-D P1	5'-CTTCATATGATGGAAGGGGAAGCTGGTTCAT-3'
JAZ12-D P2	5'-CTTCTGCAGTTATGCAACATGAGCTGGAGCT-3'
JAZ13-D P1	5'-CTTCATATGATGTCGTCTTGCTCGGAATCTAC-3'
JAZ13-D P2	5'-CTTGGATCCCTATGGTGATTGAGCAGCCAAAC-3'
MYC2 P1	5'-CTTCATATGATGACGGACTATCAGTTAGCACC-3'
MYC2 P2	5'-CTTCTCGAGCTATCTTGCATCTCCAAGTTTGG-3'
MYC3 P1	5'-CTTCATATGATGAATCTTTGGTCGGACGATAAC-3'
MYC3 P2	5'-CTTGGATCCCTATCTTGGATCTCCAACTTTGG-3'
COI1 P1	5'-GGGCATATGATGGAGGAAAATGATAGCCAAC-3'
COI1 P2	5'-CTTGAGCTCTTACAGCACCGGATCCAAAGG-3'
MYB2 P1	5'-CTTCATATGATGGCTCCAAAGAAGGCTGGAG-3'
MYB2 P2	5'-CTTCTCGAGTTATACCATTGCTAATGGATCCT-3'
MYB23 P1	5'-CTTCATATGATGGCCATGGAGGCCAGT-3'
MYB23 P2	5'-CTTGTCGACTTAGGCAAAGCCATGCCAAAC-3'
MYB25 P1	5'-CTTCATATGATGGGGAGATCACCATGTTGTG-3'
MYB25 P2	5'-CTTGGATCCTCATAAACCATTAAATCCAAAC-3'
MYB25-like P1	5'-CTTCATATGATGCAGCAGTCTCCATGTAGCG-3'
MYB25-like P2	5'-CTTGGATCCTCAAAAGACAGAAGAACCAGATG-3'
DEL65 P1	5'-CTTGGATCCACATGTCTACTGGAGTTCAACATC-3'
DEL65 P2	5'-CTTCTGCAGTCAACACTTGCTAGCAATTCTT-3'
NINJA P1	5'-GTTCATATGCATACGACGTACCAGATTACG-3'
NINJA P2	5'-GTTGGATCCTCAGGTCTGAGCAGAGCCGG-3'

Supplementary Table 2. Gene-specific primers used in RT-PCR analysis

Primer name	Primer sequence
GhJAZ1-A P1	5'-AATATCCGAACCAATTTAC-3'
GhJAZ1-A P2	5'-ATTTCCGGGTTTCGGCGGC-3'
GhJAZ1-D P1	5'-AATATCCGAACCAATGTAT-3'
GhJAZ1-D P2	5'-ATTTCCGGGTTTCGGAGGT-3'
GhJAZ2-A P1	5'-AGGATAGGATGACCACGAGG-3'
GhJAZ2-A P2	5'-GCCGATACCTAGAAACCAA-3'
GhJAZ2-D P1	5'-AGGATAGGATGACCACAAGT-3'
GhJAZ2-D P2	5'-GCCGATAACTAGAAACCAG-3'
GhJAZ3-A P1	5'-ATAGGAGCTTTAGCATCCG-3'
GhJAZ3-D P1	5'-ATAGGAGCTTTAGCATCTA-3'
GhJAZ3 P2	5'-GAGGCTCGATCCTTAATTGATG-3'
GhJAZ4-A P1	5'-TGCTTCTGTTCAGCATGATGC-3'
GhJAZ4-D P1	5'-TCTTCTGTTCAGCATGATGT-3'
GhJAZ4 P2	5'-AACATTCACGGTACCAGCA-3'
GhJAZ5-A P1	5'-ATCCTTCGGTTGAGCGACA-3'
GhJAZ5-D P1	5'-ATCCTTCGGTCGAGCGATG-3'
GhJAZ5 P2	5'-GCCTGTATCACTAAAGGTATAC-3'
GhJAZ6-A P1	5'-GCTACTGCGACTGGGAAT-3'
GhJAZ6-A P2	5'-CATTGGGTTTAGGTGGTG-3'
GhJAZ6-D P1	5'-TCTACCGCGACTGGGAAT-3'
GhJAZ6-D P1	5'-CATCGGGTTTAGGTGGCA-3'
GhJAZ7-A P1	5'-CACCCAAAGTTTTTAATAA-3'
GhJAZ7-A P2	5'-CACCGGTTCCCATCCAGTT-3'
GhJAZ7-D P1	5'-CACCCAAAGCTTTTAATCG-3'
GhJAZ7-D P2	5'-CACCGGTTCACATCCCGTC-3'
GhJAZ8-A P1	5'-CGGATGAACGACTAAGGACTC-3'
GhJAZ8-A P2	5'-GTTACTAGTAGTCATTGCTAGC-3'
GhJAZ8-D P1	5'-TCGGGATCGGGACCGGCT-3'
GhJAZ8-D P2	5'-TTAATGGTAAGGAGAGGT-3'
GhJAZ9-A P1	5'-GGCGATCTCTGCAGCAACTA-3'
GhJAZ9-A P2	5'-CATCCAACATTAATATCAA-3'
GhJAZ9-D P1	5'-GGCGATCTCTGCTGCAACTG-3'
GhJAZ9-D P2	5'-AAACATCCAACATTAATATC-3'
GhJAZ10 P1	5'-AACTCGCGGTTGAAGTTGAAG-3'
GhJAZ10-A P2	5'-CCATCCACTTGGACGGCATC-3'
GhJAZ10-D P2	5'-ACAAAACAAAATACCTCTC-3'
GhJAZ11-A P1	5'-CAAAAATGGTTGATGGCGC-3'
GhJAZ11-A P2	5'-TATTCCTCTGCACTTTGTA-3'
GhJAZ11-D P1	5'-CAAAAATGGCTGATGACGT-3'
GhJAZ11-D P2	5'-TATTCCTCTGCACTTTGTC-3'
GhJAZ12-A P1	5'-CCATCCAAAAGCTCCAGCTCAT-3'

GhJAZ12-A P2	5'-AGCCCCCTAGAGGCAGTAGG-3'
GhJAZ12-D P1	5'-CCTCAGTGCTGCAACTTCATC-3'
GhJAZ12-D P2	5'-GACAACACTCTCATGGCAAAC-3'
GhJAZ13-A P1	5'-GAATCAAGCATTGGAGTTCCT-3'
GhJAZ13-A P2	5'-ACTTCGGCTCAGCAGATTACT-3'
GhJAZ13-D P1	5'-CAAATCAAGCATCGGAGTTCCT-3'
GhJAZ13-D P2	5'-TTTGGCTCAGCAGATTACTATG-3'
GhJAZ14-A P1	5'-ATGAGGGCAAATCAAGAAA-3'
GhJAZ14-D P1	5'-ATGAGGGCAAATCGAGAAG-3'
GhJAZ14 P2	5'-GTAGCTTGGATTCTGTTC-3'
GhJAZ15 P1	5'-CGGTTTCCGTAATCAATGTACC-3'
GhJAZ15-A P2	5'-ACCAAGCATAAGGGGGAGTCA-3'
GhJAZ15-D P2	5'-CTGGTTTAGGTGGTGTATAGC-3'
GhUBI1 P1	5'-GGGATGCAAATCTTCGTGAAAAC-3'
GhUBI1 P2	5'-CTGAATCTTCGCTTTCACGTTATC-3'
